# Supplementary figures and images for: Lhx3/4 initiates a cardiopharyngeal-specific transcriptional program in response to widespread FGF signaling
Source: PLoS Biol. 2024 Jan 25;22(1):e3002169. doi: 10.1371/journal.pbio.3002169 (PMC10810493; doi:10.1371/journal.pbio.3002169)

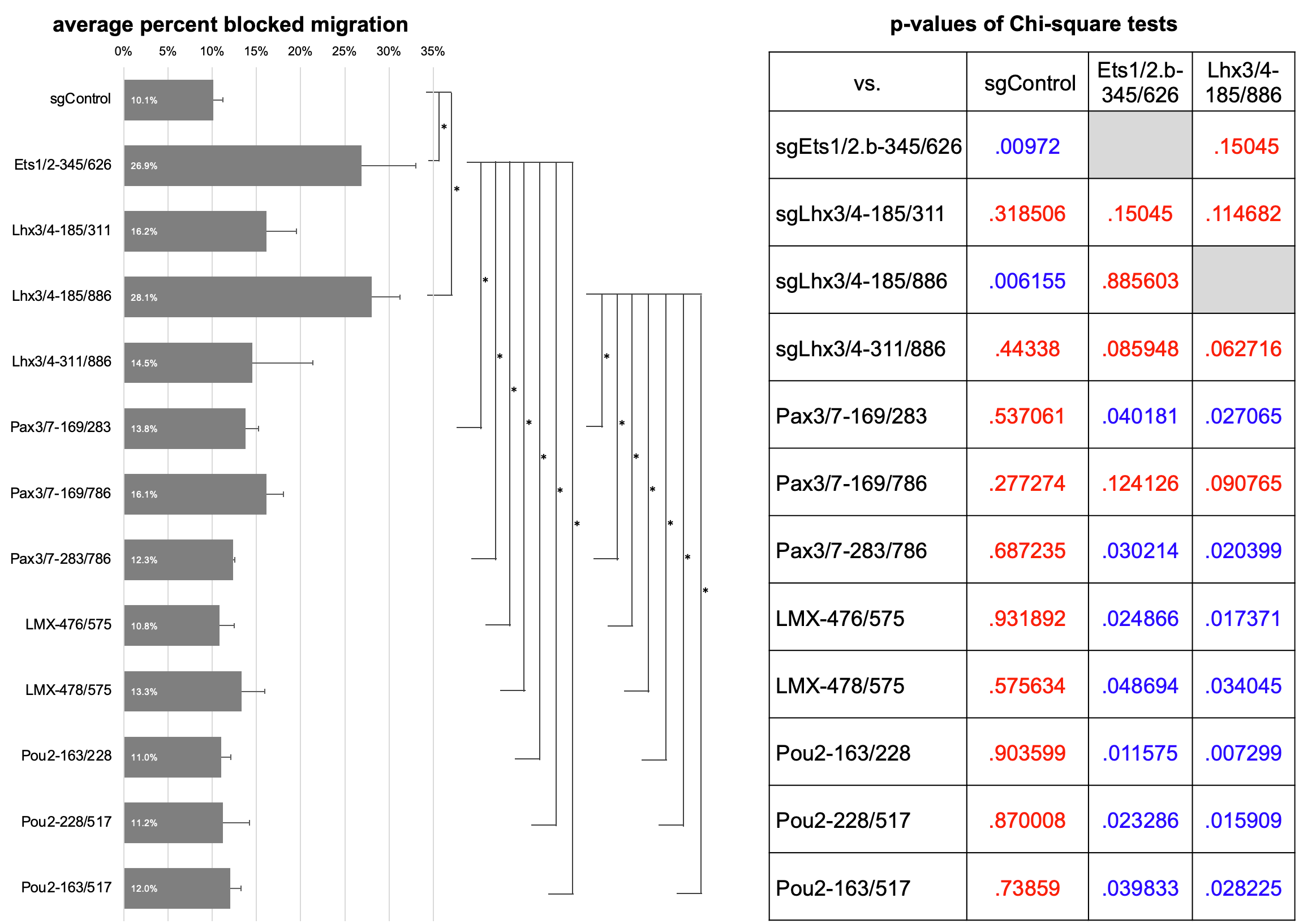

Supplement: S1 Fig — (Left) Average percent of embryos displaying perturbed migration covering 2 trials for all of the sgRNAs tested in this study (* = p < .05). (Right) Table of p-values comparing sgRNA effects on migration. Column 1) p-values for negative controls vs. other sgRNAs; column 2) Ets1/2.b sgRNAs vs. other sgRNAs; and column 3) the 185/886 pair of Lhx3/4 sgRNAs vs. other sgRNAs. (TIF) [file pbio.3002169.s002.tif]

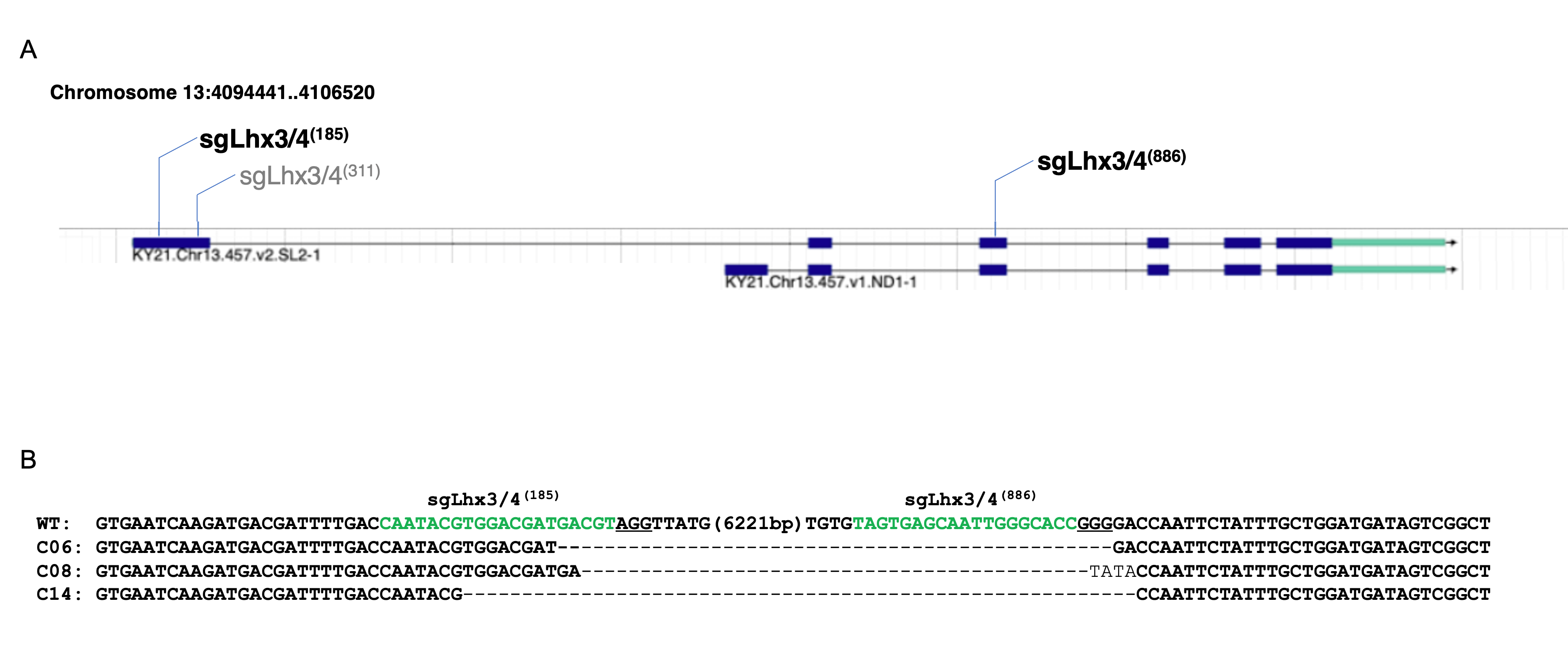

Supplement: S2 Fig — (A) Diagram of Lhx3/4 locus depicting the 2 isoforms of Lhx3/4 and the locations targeted by Lhx3/4 sgRNAs. (B) Sequencing results. WT spans upstream of the sgLhx3/4185 position to downstream of the sgLhx3/4886 position, (6,221 bp) indicates the nucleotides in the intervening wild-type sequence, underlining marks sgRNA NGGs. C06, C08, C14: sequenced clones of CRISPR morphant amplicons. Dashes indicate missing nucleotides in the sgRNA-targeted sequences. Two clones (C06/C14) had deletions, while one clone (C08) had a deletion and an addition of TATA (nonbolded). (TIF) [file pbio.3002169.s003.tif]

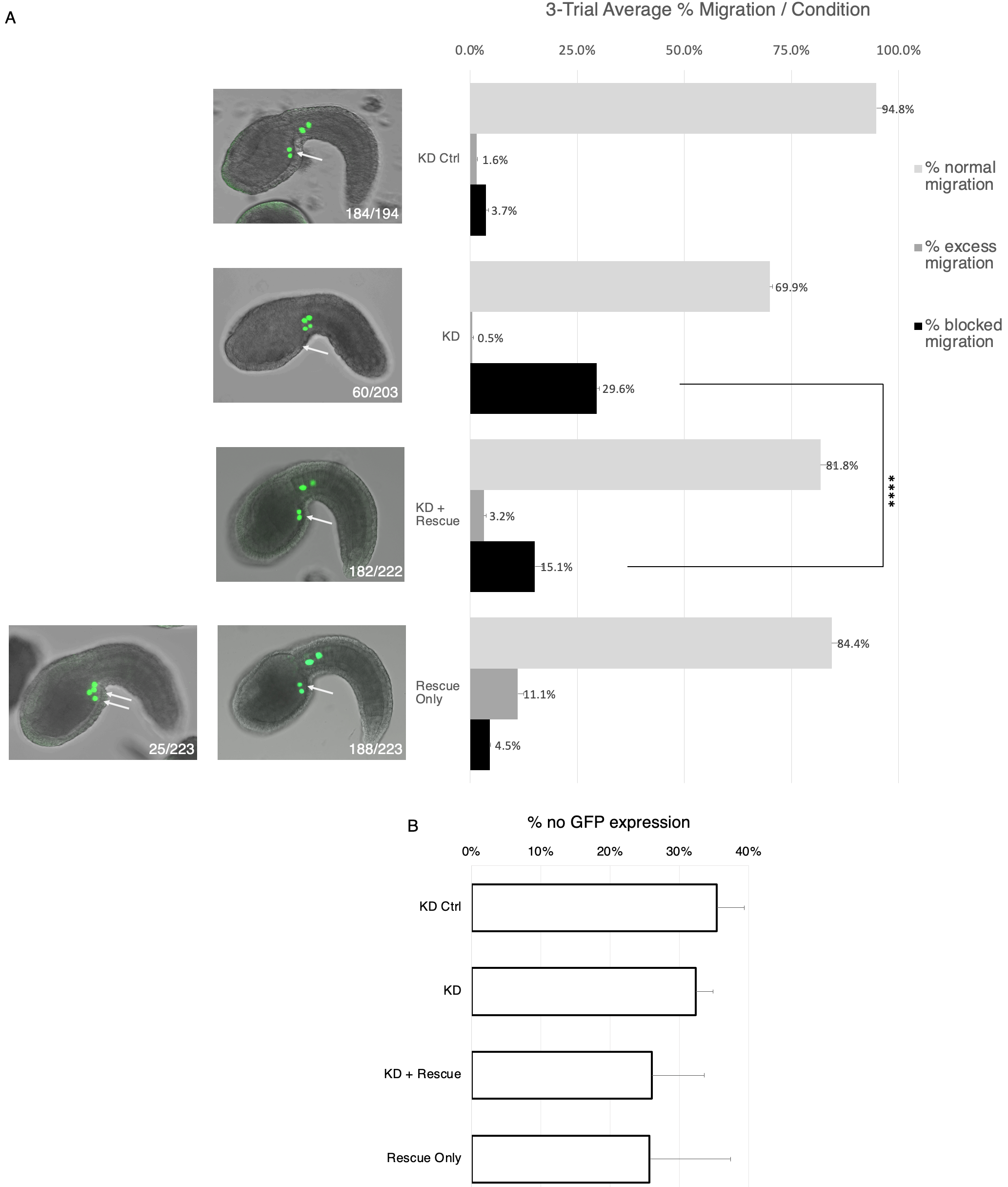

Supplement: S3 Fig — (A) Left: representative embryos, inset numbers refer to quantity of embryos displaying the phenotype presented by each image over total GFP+ embryos for each group. Arrows point to normal CPP position at this time point. Green, GFP fluorescence. Right: graph of percent migration phenotype for each group for GFP+ embryos. Blocked migration refers to CPPs in the tail; excess migration refers to CPF-lineage anterior tail muscle cells in the trunk. n = 300 per condition. **** indicates t test p-value of < .0001. (B) Graph of overall percent GFP-negative embryos per condition. (TIF) [file pbio.3002169.s004.tif]

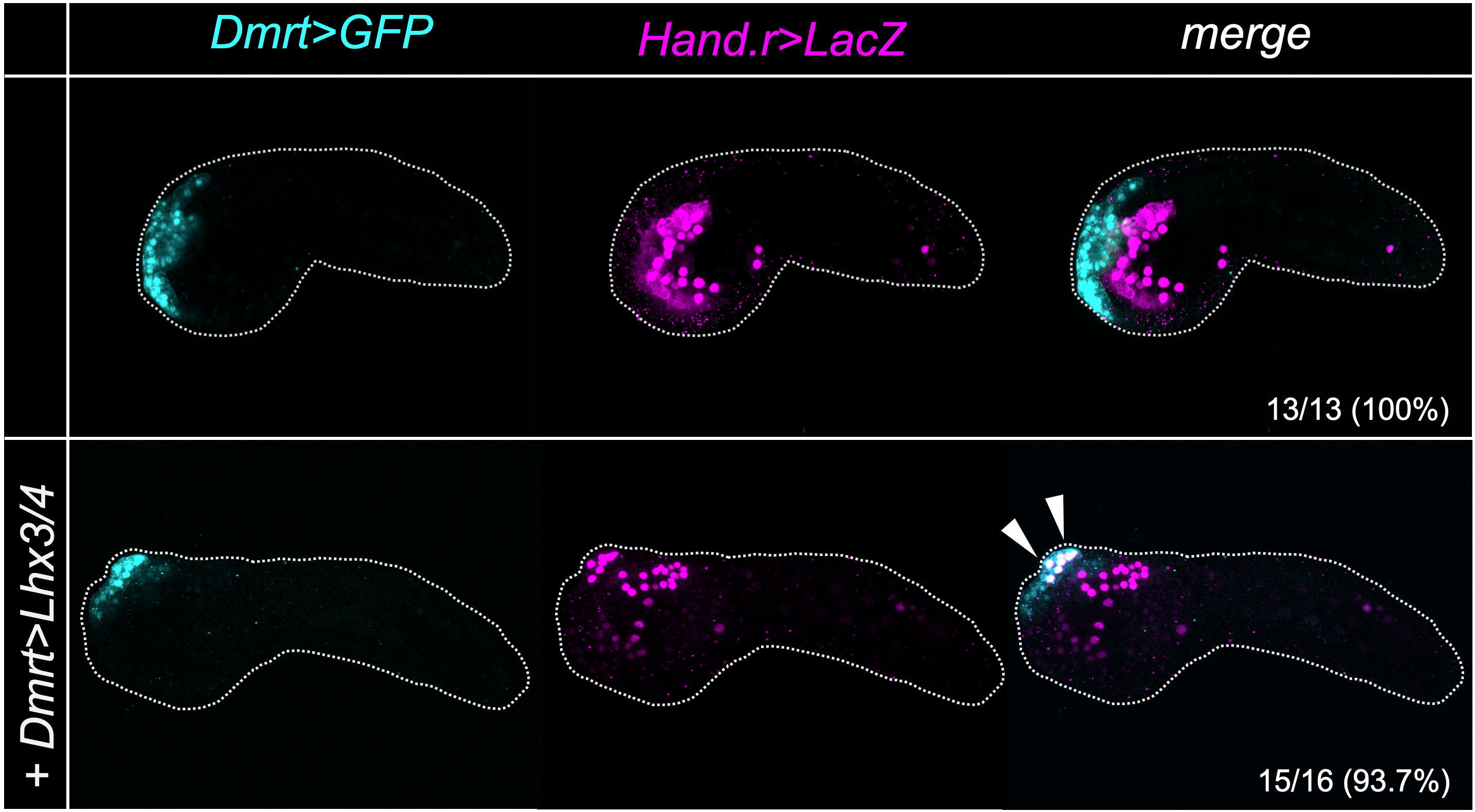

Supplement: S4 Fig — (Top) Representative control embryo electroporated with Hand.r>LacZ and Dmrt>GFP. (Bottom) Representative embryo electroporated with Hand.r>LacZ, Dmrt>GFP, and Dmrt>Lhx3/4 displaying Hand.r>LacZ reporter expression in the ANP lineage (white arrowheads). Confocal max projected Z-stacks were scored over 2 trials. In the Dmrt lineage, 13/13 (100%) of control embryos exhibited no LacZ staining, whereas 15/16 (93.7%) of experimental Hand.r>LacZ and Dmrt>Lhx3/4 transgenic embryos were positive for LacZ staining in the Dmrt lineage. (TIF) [file pbio.3002169.s005.tif]
