# Supplementary material for: Lhx3/4 initiates a cardiopharyngeal-specific transcriptional program in response to widespread FGF signaling
Source: PLoS Biol. 2024 Jan 25;22(1):e3002169. doi: 10.1371/journal.pbio.3002169 (PMC10810493; doi:10.1371/journal.pbio.3002169)
Supplement: Attachment — Submitted filename: Response to Reviewers.docx [file pbio.3002169.s016.docx]

REVIEWS:

Reviewer #1: The study by Pickett et al 'Lhx3/4 acts as a cardiopharyngeal lineage determinant, dictating lineage-specific transcription in response to widespread fibroblast growth factor signaling' proposes that a previously uncategorized subcircuit, co-factor dependent induction, is an important element of gene regulatory networks. More specifically, using the ascidian Ciona robusta, they show that the transcription factor Lhx3/4 acts as a co-factor of FGF-dependent cardiopharyngeal fate determination (cardiopharyngeal progenitor CPP induction during neurulation) downstream of its previously demonstrated function as a cell autonomous determinant of the same lineage earlier in development (cardiopharyngeal founder cell specification during gastrulation). Using tissue-specific CRISPR/Cas9 gene inactivation, they show that CPP identity and migration is impaired. Overexpression of Lhx3/4 in the anterior neural plate lineage, that is also specified in an FGF-dependent manner, is sufficient to activate the cardiopharyngeal genetic program and impairs neural tissue morphogenesis and differentiation.

This study, the underlying subcircuit and the theoretically implications are of great importance and potentially very interesting for a large readership. However, the primary data that are presented here suffer from a number of caveats that prevent a robust support of the model.

**We thank this reviewer for their appreciation of the importance of this work and their careful review and numerous substantive comments!**

Major points:

1) The manuscript contains a number of mistakes and imprecisions (listed in the minor points) that make the reading difficult and question the thoroughness of the study.

**We thank the reviewer for catching these minor errors and have corrected all of them as detailed below.**

2) General considerations on CRISPR/Cas9 gene experiments. There are a number of details to clarify regarding the method, the evaluation of the mutagenesis and whether mutations produce loss-of-function.
        - What is the control condition? Empty vector (line 187, Fig 2) or a sgRNA against GFP (line 228, Fig 3)? What is the quantity of plasmid that is electroporated?

**We agree that precision in describing reagents and controls is extremely important. We have added text to clarify the exact reagents and controls used in each experiment including empty vector controls for the CRISPR experiments and we have listed quantities of plasmids used in supplemental table 1. Additionally, since all sgRNAs tested were electroporated in the same experiment, negative results from Pou2, Pax3/7, and LMX sgRNAs serve as additional controls. We also performed a new rescue experiment by expressing a sg-RNA resistant form of Lhx3/4, and showed that this resulted in a significant restoration of migration (Figure 2F-H).**

        - What are the actual sgRNAs that have been used and lead to a phenotype (see minor points)? **We have modified the text to make clearer where readers can find this information. All sgRNA sequences are listed in Supplemental Table 1. Furthermore, we have added sgRNA CRISPOR scores and all data regarding the impact of CRISPR on CPP migration for each sgRNA combination tested (Supplemental Figure 1 and Table 1).**

- A 16% indel frequency is shown in Fig 2F. However, since pairs of sgRNAs have been used, one expects to obtain deletion of the piece of chromosome between the 2 sgRNAs or indels at both sites. This is not shown.

- Since the pair of efficient sgRNAs is unclear, one is left to imagine. From my own search, there are 2 possible scenarios. In the first case, 1 sgRNA targets the 5'UTR region and the other the coding sequence. The PCR designed to amplify the 5'UTR is thus irrelevant. In the second case, both sgRNAs target the 5'UTR. The PCR and sequencing are relevant but from the data, it seems that only one sgRNA is efficient, and this should be explicit. In addition, the authors should explain how, in this specific case, 5'UTR mutations would correspond to a loss-of-function.

**We thank the reviewer for pointing out this oversight. It is true that we used a pair of sgRNAs that target two chromosomal locations at a distance from one another. We have included a new diagram showing sgRNA(Lhx3/4) locations (Supplemental Figure 2). We have also added text to make clear exactly where sgRNAs targeted, why those locations were chosen, and shown the sequencing results covering the repaired junction (Supplemental Figure 2).**

 - For all targeted genes, the genomic locus (with gene/transcript/ORF) and sgRNAs positions should be displayed. Also, the mutation efficiency (and loss-of-function) should be evaluated.

**As mentioned above, we have added CRISPOR scores to Supplemental Table 1 and we have included data regarding loss of function impacts for all sgRNAs in supplemental figure 1. We have also diagrammed the genomic locus and sgRNA positions. We have also clarified that the number give to each sgRNA (e.g., Lhx3/4(185) means that that sgRNA targets the 185^th^ nucleotide of the coding sequence. We also provided the exact sgRNA targeting sequences which all together should give readers sufficient information to easily understand and/or repeat the experiments.**

3) A difficult issue is to distinguish the early and late functions of Lhx3/4. The CRISPR-mediated LOF is targeting the late FGF-dependent function. There are 2 main supporting evidence provided by the authors: the use of the Mesp enhancer and the lack of effect on Ets1/2.b expression. It would be important to further support the fact that early function is not impaired. I suggest the following experiments. The authors should make sure that Mesp expression is not affected: either by in situ hybridization of the gene itself or by scoring the proportion of GFP positive cells or embryos in the experiments shown in Fig 2. The authors should also provide a rescue for the experiment in Fig 2.

**To further buttress this interpretation, we scored the proportion of Mesp>H2B::GFP positive samples as suggested. This data is included in supplemental figure 3b.**

Finally, by expressing Cas9 using an earlier driver, it should be possible to block the early function of Lhx3/4, and the phenotype should be different (loss of Mesp progenitors).

**We appreciate this suggestion, however early knockdown of Lhx3/4 has previously been published (**<https://doi.org/10.1016/j.ydbio.2009.01.033>) **and, along with suppressing Mesp expression, these manipulations led to severe disruption of early development due to the function of Lhx3/4 in endoderm development. Unfortunately, there are no early drivers that are specific to the founder cell lineage, thus this issue cannot be resolved in order to productively compare early vs. late knockdown of Lhx3/4 as recommended. As discussed in the manuscript, it is unlikely that Mesp itself could be knocked out using the Mesp driver. Additionally, we show no impact on Ets1/2 expression and on Mesp reporter expression (thanks to the suggestion of this reviewer) and we also show that Mesp-driven expression of a sgRNA-resistant form of Lhx3/4 restores migration. Thus the evidence we provide to support our interpretation is very strong.**

4) Results in Fig 4 are exciting but should be expanded to robustly support the model proposed by the authors.
In Fig 4I it seems that the FoxF reporter is expressed in 3 rows of cells of the neural plate suggesting that it may also be expressed in the palp precursors. Since this territory is supposed to have silent FGF signaling (Wagner et al, 2012), the results do not fully match the model. I would thus recommend to accurately determine the reporter expression within the neural plate. It would also be interesting to determine FoxF (or other cardiopharyngeal genes) expression directly by in situ hybridization.

**This comment was very helpful and encouraged us to take an ambitious approach to expand on this experiment as recommended by the reviewer. In particular, we conducted mRNA seq on sorted ANP cells confirming ectopic expression of Foxf and other CPP genes and also leading to a number of additional, intriguing findings (Figure 6 and associated text). We also added text to the discussion regarding the expansion of ectopic CPP reporter activity into all four rows of the ANP included the more anterior palp region that is normally subjected to repression of FGF-dependent gene expression.**

Next, it is essential to show that launching the cardiopharyngeal program is FGF dependent. The authors could perform the same experiment as in Fig 4I in embryos where FGF signaling is inhibited at the adequate stage. The authors could also express Lhx3/4 in the epidermis where FGF signaling is not active, and test whether activating the FGF pathway triggers a different response.

**Thanks for this excellent suggestion. As recommended, we performed the Lhx3/4 misexpression experiments in the presence of an inhibitor of the MapK pathway downstream of FGF - U0126 (Fig 4). The reviewer will see that our results mostly aligned with our current model but that there was an interesting exception that suggested some new potential insights.**

5) To further support the relevance of their model, it would be important to provide another example (not only the ectopic overexpression of Lhx3/4). I would suggest to drive Gata.a or Zic-r.b expression in the cardiopharyngeal lineage (using Mesp driver?) and determine the FGF-dependent consequences.

**Although we appreciate that this would be an excellent complementary set of experiments to perform, the paper already includes extensive data and novel findings, including newly performed RNA sequencing to fully examine the impact of Lhx3/4 misexpression. To fully explore the impact of GATAa or Zic-r misexpression would require extensive further research that is beyond the scope of this initial effort and would have been impossible for us to conduct during the limited time window provided for revision. We do plan to complete these experiments for a follow up study.**

6) The authors have focused on cell behavior and morphogenesis following Lhx3/4 overexpression. It would also be interesting to look at the neural plate genetic program.

**This was another great suggestion and we have addressed it through analysis of RNA sequencing data, revealing extensive downregulation of the native ANP genetic program following Lhx3/4 overexpression (Figures 6D, E).**

Minor points:

- Ets1/2.b being a direct target of Mesp is a critical element for the model and for the data in Fig 3F-H. Could the authors describe the supporting evidence?

**Although direct regulation of Ets1/2b by Mesp has been assumed to occur in various reviews and prior articles, the experimental evidence supporting this has not been published and therefore we were not able to cite it. In summary, knockdown of Mesp leads to loss of Ets expression and mutation of putative Mesp binding sites in the Ets enhancer leads to loss of enhancer activity. We hope that the paper including this data (which was performed with a collaborator) will be published soon. In the meantime, we used a dotted lines to depict this regulatory connection since it has not been formally demonstrated.**

- line 107: Ciona intestinalis b is uncommon. Should be Ciona intestinalis type B. A reference on the two types is missing. **This has been corrected to indicate that this is Type A – which is Ciona robusta.**
- line 200: 'this pair'. Which one is it? **This has been addressed**
- Figure 2F: indicate sgRNA position and name. **This has been addressed, see detailed comments above – and supplemental figure 2.**
- Figure referencing:
        line 129: should be 1C.
        line 299: 3O does not exist.
        lines 385-386: should not be 6C and 6D,E.

**These errors were corrected**

        supplemental table 1: it is not clear which experiments/figures the upper part of the table refers to.

**All experiments/figure panels are now indicated.**

- Unpublished/not shown data:
        line 125 Mesp activates Ets1/2.b
        line 472 Pigmentation

        line 643 Foxf epi enhancer deletion

**We have addressed questions about Ets1/2 regulation above and modified the text to preclude any mention of unpublished data in the other two instances.**

- Inconsistencies between Supplemental Table 1 and Supplemental Fig 1:
        sgRNAs against Ets1/2.b do not have the same names. **This has been corrected, the correct sgRNAs used have been added to the table**

        sgRNA LMX-478 is not listed in the table **This has been added**
Which sgRNAs have been actually used? **This has been addressed, see Supplemental table 1**
- Supplemental Fig 1. Why statistical tests have been conducted with the sgRNA combination against Lhx3/4 that has the weakest effect? **There was a typo in that table. Statistics were performed against the “185/886” Lhx3/4 sgRNAs (which generated the highest effect)**
- Fig 3H: the legend should be % Ets1/2.b expression. **This has been corrected**
- Could the authors comment on the higher penetrance of the Lhx3/4 CRISPR phenotype in juveniles compared to embryos?

**This may relate to changes in the level of expression for CPP genes which still may have resulted in defective heart formation even though we would not have scored them in the previous assays. We did not add this comment into the manuscript, but we are open to doing so if the reviewers or editors feel that this is would be preferrable.**

Reviewer #2: Pickett et al have investigated the role of Lhx3/4 in development of the cardiopharyngeal lineage in Ciona. The results of this clearly written and carefully documented study suggest that Lhx3/4 plays an important lineage determining role in cardiopharygneal cell migration and heart development. The authors argue that this is consistent with a role as a partner of FGF signal dependent Ets transcription factors, addressing the important question of how gene regulatory networks integrate extrinsic and intrinsic inputs. However, the following points appear necessary to reinforce the authors conclusions.

**We deeply appreciate this reviewer’s assessment regarding the importance of this study and their insightful comments and suggestions.**

1. The authors convincingly show that Lhx3/4 is required for CPM migration, Hand gene expression and heart morphogenesis, and phenocopies Ets1/2 knock-down. These results are consistent with the authors' underlying hypothesis. However, in my reading of this manuscript it seems that these experiments do not directly demonstrate that Lhx3/4 is an Ets cofactor. Or that the role of Lhx3/4 is to confer lineage specific transcription in response to FGF signaling. As FGF signaling is also required for these phenotypes it is clearly challenging to show the FGF-signal dependence of Lhx3/4 function. Could the authors demonstrate molecular or genetic interactions between Ets1/2 and Lhx3/4 that would reinforce their interpretation? Can they rule out that Lhx3/4 plays FGF-independent roles in regulating cardiopharyngeal lineage migration? This should at a minimum be discussed and in the absence of such evidence the conclusions should be tempered.

**This is an excellent point. As detailed in our response to Reviewer 1, we have conducted experiments demonstrated that Lhx3/4 functions in tandem with FGF/MapK signaling to drive CPP expression (in the context of the gain of function experiments). Interactions between Ets1/2 and Lhx3/4 are not required for them to function as co-factors (they could bind independently to respective sites and drive expression synergistically without any direct interaction with each other). Prior experiments have also already provided extensive evidence that both Ets and ATTA binding sites are required for FGF-dependent induction of CPP gene expression. We have also added language to clarify the need for further experiments to the discussion… “**It will also be critical to further assess the exact contributions of Ets1/2 and Lhx3/4 both in regulating initial autonomous CP founder cell specification and in subsequent non-autonomous regulation of CP progenitor specification and to examine whether these two factors directly interact or function in an independent manner during each of these regulatory steps.**”**

2. This point also applies to the very interesting ectopic expression experiments that confer cardiopharyngeal migratory phenotypes and disrupt neuroectoderm development. Can the authors show that this is really through ectopic partnering with Ets factors as opposed to FGF-independent roles of Lhx3/4 in driving a cardiopharyngeal program? For example, can the authors show the Lhx3/4 phenotype is dependent on Ets1/2 or FGF signaling in the neurectoderm? Or could they show that ectopic Lhx3/4 activation of the Foxf or Hand enhancers is dependent on the Ets1/2 binding sites? In the absence of such evidence it might be more prudent to present the FGF signal partner role as one possible mechanism of Lhx3/4 function.

**This is an excellent point.** **As detailed in our response to Reviewer 1, we have conducted experiments demonstrated that Lhx3/4 functions in tandem with FGF/MapK signaling to drive CPP expression.**

3. Do the authors know if Lhx3/4 is involved in iterative roles at each step of CPM FGF signal response, through to activation of the pharyngeal muscle lineage? Are pharyngeal muscles also perturbed after Lhx3/4 inactivation? Discussion of the established role of Lhx2 in mammalian cardiopharyngeal mesoderm would be pertinent (PMID: 23112163).

**Another great point! We did not investigate the potential impact of our manipulation on pharyngeal development but as the reviewer points out, since the pharyngeal muscles are also derived from the CPPs, and because there are subsequent steps in CPP sub-specification that involve FGF, this would be an excellent focus for a follow up study. We added a sentence to the discussion in regards to the article on mammalian Lhx2, and are grateful to the reviewer for bringing this to our attention. “**Although previous studies suggest that Lhx2 participates in cardiac and craniofacial development in mouse embryos (PMID: 23112163) further studies will be needed to assess potential evolutionary implications regarding cardiopharyngeal development within the shared tunicate/vertebrate ancestor.”

4. The introduction and discussion could be significantly shortened. Some of the GRN points would be more suitable for a review, including parts or all of Figure 6, which is somewhat speculative, especially given points 1 and 2 above. If the Lhx3/4 role can be shown to depend on coincident FGF signaling this may be more relevant. I

**We appreciate this suggestion. Since we now have shown that FGF/MapK signaling is required for ectopic activation of CPP gene activity we feel that the current introduction and discussion are appropriate. We have also moved our models out of the results section and condensed them into a single figure associated with the discussion. Additionally, some of this material is focused on pointing out that current models are inadequate and our attempts to begin addressing these gaps on a theoretical level. We think these are important points as summarized in the third reviewer’s summary.**

Reviewer #3: The paper nicely shows that Lhx3/4 is an instructive co-factor for FGF/MAPK-induced Ets transcription factor (TF) in the Ciona cardiopharyngeal (CP) lineage. This is convincingly shown with proper experiments, adapted controls and sufficient statistical evluation.
The originality lies in the conceptualization into a more broadly applicable gene regulatory subcircuit that takes into consideration the prerequisit of the signaling pathway activation of the TF (Ets) in addition to its mere presence. Such Ets pathway activation is then alteratively combined with different lineage determining TFs for tissue specific effector gene activation. While such combinatorial code was previuosly shown for other lineages the experimental proof of ectopic instructive behaviour and consequent 'confused' cellular morphology pinpoints this concept. In addition, Lhx assures a cascadal feed-forward loop in the CP lineage through two subsequent steps in synergistic activity with different co-factors whereby the later co-factor (Ets) is a consequence of Lhx early function (Lhx activating Mesp transcription that causes Ets transcription).
The conceptualization of a general inducible TF (Ets) activation principle in combination with different lineage determinants is important to a wider scientific community, also outside Ciona, notably because MAPK/Ets signaling is pleiotrophic and highly conserved. A differentially interpreted synergism with lineage determinats may generally explain how tissue specificity is achieved, also in the human cell differentiation and disease context.

**We greatly appreciate this reviewer’s assessment of our manuscript and in particular for supporting our efforts to make broader points regarding critical gaps in current models.**

Overall the paper is properly written, built and is adequately taking into account the literature etc. and can be recommended for publication -
however, given that the following suggestions are taken into account.

The authors test a few candidate 'ATTA' binding TFs by CRISPR in the CP precursors (CPP) and these candidates were supposedly a result of their previous study. More precise support should be given why these TF are actual candidates (expression patterns etc.).

**We thank the reviewer for this recommendation and have clarified the nature of this previous work. “**The *C. robusta* genome contains eighty-six homeobox TFs [51]. However, a previous study revealed that only a small subset of these factors are enriched in sorted cardiopharyngeal founder lineage that were subjected to expression analysis just prior to CPP specification (Figure 1A, B) [35].”

Only Lhx CRISPR was successful but no indication was given that the guides/CRISPR for the other TFs actually worked. Should the authors have genotyping data for these other TFs it would strengthen the uniqueness of the Lhx effect. If not they may at least state that this is still an open question.

**We appreciate this comment and have added the following statement to the relevant results section. “**Because we did not conduct further analysis of sgRNA knockdown for the other targets, we cannot rule out a potential role for one of these alternative candidate factors in CPP specification.”

There are sections in the Results that should go in the Discussion as they interrupt the flow of data and don't actually contribute. This minimally concerns Figure 4B-D and associated text in lines 312 to 320 except for the rather important sentence of lines 317 to 318.

**We agree with this comment and have moved these sections of the results and associated figure panels to the discussion and final figure.**

Furthermore, the Figure 6 should be compacted in A and B (and corresponding sections in Discussion lines 510 to 524) around the putative co-factor Dlx (that may also bind ATTA motifs) and should leave out consequent speculations about lineage specific activators and repressors. Either the authors bring the actual proof that Dlx functions this way and then speculate how the embryo sorts out two factors that both bind to ATTA motifs or they just leave that out since there may be another mechanism at work than speculated. This simplifies the message and streamlines it to what they have actually shown.

**Although we appreciate this suggestion we have chosen to leave in this section of the discussion as it addresses an important theoretical implication of our model. In particular, along with dealing with potential deployment of co-factors that bind the same sites, these alternative models are also used to discuss whether cofactors function to instruct or facilitate differential gene expression and to help us interpret our gain of function results in association with these two distinct, hypothetical circuits.**

More minor points and typos:
Line 102: C. robusta is C. intestinalis Type A (not B) **This has been addressed**
Figure 2A: Please also include the older KH nomenclature, for easier comparison to previous studies **While we agree it may be ideal to have both models presented for all genes discussed, the set of genes referenced in this study has expanded greatly. We therefore see this addition as impractical and have added the suggestion to readers in the Figure 2 legend that they can simply go to the new version of the GHOST website (**[**http://ghost.zool.kyoto-u.ac.jp/default_ht.html**](http://ghost.zool.kyoto-u.ac.jp/default_ht.html)**) and quickly convert particular genes of interest manually.**

Line 360: change CFP to CPP **This has been addressed**

Figure 5C and E: DMRT>GFP would be an appropriate control, not Mesp>GFP.

**We agree this would be a more appropriate loading control but the use of Mesp>GFP does balance out the overall mass of construct used in control vs. experimental samples and still consitutes a highly effective control group. We chose to focus our efforts in revision on conducting an extensive set of new experiments instead of repeating this one with a new control.**

Lines 381-382: Figure 5 not 6 **This has been addressed**
Line 406: eliminate the braquet + text **This has been addressed**

Lines 436-446: correctly associate Figure 5 labels in the text and in the legend (H to L) **This has been addressed**
Line 629: may be BsaI not BsmBI **This has been addressed.**
Supplement Table 1: correct to Figure 2A-F and Figure 3I-N (not 2) **This has been addressed**
