# Supplementary material for: Lhx3/4 initiates a cardiopharyngeal-specific transcriptional program in response to widespread FGF signaling
Source: PLoS Biol. 2024 Jan 25;22(1):e3002169. doi: 10.1371/journal.pbio.3002169 (PMC10810493; doi:10.1371/journal.pbio.3002169)
Supplement: Attachment — Submitted filename: 2nd Response to Reviewers.docx [file pbio.3002169.s017.docx]

**Editor Remarks:**

Based on the reviews, I am pleased to say that we are likely to accept this manuscript for publication, provided you satisfactorily address the remaining points raised by the reviewers. This includes adding the additional reporting details requested by Reviewer's #1 and #3 and shortening the Introduction and discussion sections as suggested by Reviewer #2.

**We have added these details and shortened the introductory and discussion sections as requested.**

In addition, I would be grateful if you could please make sure to address the following data and other policy-related requests that I have provided below (A-E):

(A) We would like to suggest the following modification to the title:

“Lhx3/4 initiates a cardiopharyngeal-specific transcriptional program in response to widespread FGF signaling” **We have changed the title as requested. While we feel that our original title better captures this manuscript’s major implications (as mirrored in our discussion), we are open to using your suggested title.**

(B) You may be aware of the PLOS Data Policy, which requires that all data be made available without restriction: <http://journals.plos.org/plosbiology/s/data-availability>. For more information, please also see this editorial: <http://dx.doi.org/10.1371/journal.pbio.1001797>

Thank you for already providing the individual numerical values that underlie the summary data displayed in the figures (Appendix S1 file). However, it seems that the figures specified in this document do not match the figure panels that we would require underlying data for, which are as follows:

**We feel your helpful suggestions and requirements are clearly stated, and have made the appropriate changes to the following three notes. Please let us know as soon as possible if we have misinterpreted any of your requests.**

Figure 2B, 2F, 3D, 3H, 3L, 4G, 4J, 4O, 5D, 5K, 6B, S1, S3A-B **(This has been addressed)**

We would be grateful if you could double check the Appendix S1 file and edit the tabs if needed. In addition, we note that the Appendix S1 file does not include underlying data for the supplementary files (S1 and S3A-B) and we ask that this is included at this stage. **(This has been addressed)**

NOTE: the numerical data provided should include all replicates AND the way in which the plotted mean and errors were derived (it should not present only the mean/average values). **(This has been addressed)**

(C) Thank you for uploading the mRNA-sequencing datasets in the Appendix S2 file. Given the potential size of the dataset, we would encourage you to deposit this data in the GEO repository and provide the accession number in the Data Availability Statement of the online submission form. **We have uploaded all raw and processed data to the GEO depository. We have provided the accession number in the appropriate area in the submission form.**

(D) Please also ensure that each of the relevant figure legends in your manuscript include information on *WHERE THE UNDERLYING DATA CAN BE FOUND*, and ensure your supplemental data file/s has a legend. **We find this requested feature helpful, and have added this notation in each figure legend to each instance where relevant.**


(E) Please ensure that your Data Statement in the submission system accurately describes where your data can be found and is in final format, as it will be published as written there. **We have reworded this section in the submission system and believe we have effectively communicated locations for all relevant data.**

**Reviewer remarks:**

**Reviewer #1**: I am happy with the revised version of the manuscript that I find of much better quality. **We agree the manuscript is much improved, and thank the reviewer for your helpful comments, suggestions, and the thoroughness of the reviews.**

There are some minor points that deserve corrections/clarifications:
- lines 236-238: should be Figure S2 **Thank you for bringing this typo to our attention, it has been addressed.**

- Table S1 is missing from the submission **Thank you for pointing out the absence of this document, it should have been contained in the V2 document and we apologize for this accidental omission. It is included in this current revision.**

- Fig S2B:
        The sequence of sgLhx3/4(185) does not correspond to the sequence present in the 1st submission and to the displayed sequence of the mutant clones (1 SNP). **We appreciate you correctly pointing out that the sequences from one version to the next do not match: the SNP you reference is a typo, which we have fixed. It appears the “WT” sequence (**CAATA**T**GTGGACGATGACGTAGG) **we copied from the KY21 browser when making the figure, is different than 1) the KH2012 version, 2) our sanger-sequenced amplicons, and 3) the actual sgRNA sequence used in our experiments (**CAATA**C**GTGGACGATGACGTAGG). **We sincerely appreciate the reviewer’s degree of thoroughness.**

        The sgLhx3/4(886) size/PAM do not fit either. **Thank you for pointing out this typo, which we have addressed. The sequences are all correct, however, the issue was an accidental leftward shift in the underlining of the PAM sequence in the diagram of Figure S2, which made it appear as if there were significant differences in the sgRNA sequences described and the chromosomal location. We also recognize that the sgRNA sequence is not present in any of the sequenced amplicons presented in Figure S2. However we suggest that this was due to NHEJ repair errors which often lead to deletion of some or all of the sgRNA targeted sequence (see Ghandi 2017; PMID 28341547).**

        The displayed mutant clones seem different from the ones shown in Fig 2F of the 1st submission. **This is a correct observation. We completely reperformed the experiment, and we should have been clearer about this change in our first reviewer response. The sequencing in the first experiment utilized an oligo pair that would only amplify a single indel caused by only sgLhx3/4^185^, whereas in the revision, we resorted modified CPP cells and reamplified the full target region using an oligo pair designed to amplify the region between the two targeted sites. We have modified the methods section to more clearly describe this approach.**

        The frequency of mutant clones is not indicated. **Because we amplified the full target region from pooled cells it was not feasible to compare the incidence of mutant vs. non-mutant clones because these would be represented by distinct amplicons. Additionally, we only obtained amplicons when there was a deletion in between the two target sites. Therefore we do not have any data on the frequency of mutant vs. non-mutant clones.**

- The RNAseq data should be deposited in a public repository, and the accession number provided in the manuscript. **We have deposited all raw and processed RNAseq data and the accession information will be included in the manuscript.**

- RNAseq data were analyzed using KH2012 gene models, but KY21 gene models are shown in Fig6. Please indicate how the conversion was made. **We added an additional note in the M&M to clarify this further “Referenced KY21 genes were manually converted using the KY genome browser (Satou 2022). KH IDs were input into the genome browser search bar. Results on the genome browser contained the KY IDs and homolog information.”**

**Reviewer #2**: The authors have extensively revised their nice manuscript in the light of the earlier comments. This includes addition of important experiments demonstrating that ectopic Lhx3/4 fails to induce the CPP program in the neural plate when the MAPK pathway is blocked. This study brings a number of new findings and directions with respect to how regulatory inputs are integrated during development. The introduction and discussion remain long and could potentially be shortened. **We thank Reviewer 2 for their original comments and suggestions, and their support of the current version. We have removed some text from the introduction and discussion as requested.**

**Reviewer #3**: Most of my concerns were addressed/explained except for the following:
1.      I could not find Supplement Table 1. It is important to list the sequences of all guides for the different genes used in this study - notably as some of them were not extensively studied **We thank the reviewer for pointing out the unfortunate absence of this document. We agree it is important to include this information and have made sure this document has been included with this resubmission. We appreciate all of the suggestions and comments Reviewer 3 has made, and typos caught, in their thorough reading.**

2.      The KH and KY21 nomenclature mix is still irritating - there is no match between Figure 6 and the corresponding lists in Appendix S2, this needs to be adjusted: please add the KY21 names and human orthologs in the Appendix S2 list as of Figure 6. **We appreciate that it is challenging to navigate these different gene IDs. To overcome these challenges we have included tabs for each relevant figure panel in Appendix S2 and revised this table to show matches between KH and listed KY gene IDs. We have revised the figure legend for Appendix S2 to more clearly explain what is shown and which gene ID numbers were used in this analysis.**

3.      For Figure 6 the highlighting in bold and color coding (Fig.6G notably) needs to be clarified in the legend **We thank the reviewer for this helpful suggestion. We have added text to the legend clarifying the bolding and color-coding as requested.**

4.      Lines 236 and 238: Figure S4 and S5 should be Figure S2 A and B **We thank the reviewer for pointing out these two typos, and have made the corrections.**

5.      Figure S3 B scoring of negative embryos is not meaningful - or explain why negative rather than positive embryos are scored (as requested by Reviewer 1 point 3); in the text (line 249) Figure S2 should be changed to S3 **We have corrected this typo and thank the reviewer for pointing it out. Regarding the first point, we have added the following text to the results section to clarify the relevance of these results – “**Notably, knockdown of *Lhx3/4* did not decrease expression of the *Mesp* reporter (*Mesp>H2B::GFP*), indicating knockdown of *Lhx3/4* did not interfere with initial CPF specification (Fig S3B).**” Comparing the negative results (which are just the flip side of the positive results) supports our assertion that Mesp reporter expression did not decrease.**
